# The Geographic Distribution of *Loa loa* in Africa: Results of Large-Scale Implementation of the Rapid Assessment Procedure for Loiasis (RAPLOA)

**DOI:** 10.1371/journal.pntd.0001210

**Published:** 2011-06-28

**Authors:** Honorat Gustave Marie Zouré, Samuel Wanji, Mounkaïla Noma, Uche Veronica Amazigo, Peter J. Diggle, Afework Hailemariam Tekle, Jan H. F. Remme

**Affiliations:** 1 African Programme for Onchocerciasis Control, World Health Organization, Ouagadougou, Burkina Faso; 2 Research Foundation for Tropical Diseases and Environment, Buea, Cameroon; 3 Department of Biochemistry and Microbiology, University of Buea, Buea, Cameroon; 4 Institute of Infection and Global Health, University of Liverpool, Liverpool, United Kingdom; 5 Consultant, Ornex, France; Centre Suisse de Recherches Scientifiques, United States of America

## Abstract

**Background:**

Loiasis is a major obstacle to ivermectin treatment for onchocerciasis control and lymphatic filariasis elimination in central Africa. In communities with a high level of loiasis endemicity, there is a significant risk of severe adverse reactions to ivermectin treatment. Information on the geographic distribution of loiasis in Africa is urgently needed but available information is limited. The African Programme for Onchocerciasis Control (APOC) undertook large scale mapping of loiasis in 11 potentially endemic countries using a rapid assessment procedure for loiasis (RAPLOA) that uses a simple questionnaire on the history of eye worm.

**Methodology/Principal Findings:**

RAPLOA surveys were done in a spatial sample of 4798 villages covering an area of 2500×3000 km centred on the heartland of loiasis in Africa. The surveys showed high risk levels of loiasis in 10 countries where an estimated 14.4 million people live in high risk areas. There was a strong spatial correlation among RAPLOA data, and kriging was used to produce spatially smoothed contour maps of the interpolated prevalence of eye worm and the predictive probability that the prevalence exceeds 40%.

**Conclusion/Significance:**

The contour map of eye worm prevalence provides the first global map of loiasis based on actual survey data. It shows a clear distribution with two zones of hyper endemicity, large areas that are free of loiasis and several borderline or intermediate zones. The surveys detected several previously unknown hyperendemic foci, clarified the distribution of loiasis in the Central African Republic and large parts of the Republic of Congo and the Democratic Republic of Congo for which hardly any information was available, and confirmed known loiasis foci. The new maps of the prevalence of eye worm and the probability that the prevalence exceeds the risk threshold of 40% provide critical information for ivermectin treatment programs among millions of people in Africa.

## Author Summary

Loiasis is a neglected tropical disease caused by infection with the filarial parasite *Loa loa*, transmitted by *Chrysops* vectors. Loiasis has recently emerged as a disease of public health importance when neurologic serious adverse events (SAEs) were reported in individuals with high *L. loa* microfilaraemia after ivermectin treatment. This had a negative impact on the control of onchocerciasis and lymphatic filariasis in areas of co-endemicity with loiasis. Microfilarial prevalence of 20% has been suggested as the threshold above which there is an unacceptable risk of SAEs with ivermectin treatment.The African Programme for Onchocerciasis Control (APOC) undertook large scale mapping of loiasis in 11 potentially endemic countries using a rapid assessment procedure for loiasis (RAPLOA) that uses a simple questionnaire on the history of eye worm. A geostatistical analysis method called kriging applied to the results in 4798 sampled villages generated a contour map of eye worm prevalence, providing the first global map of loiasis based on actual survey data. This map showed high risk levels of loiasis in 10 countries where an estimated 14.4 million people live in high risk areas.

## Introduction

Loiasis is a neglected tropical disease caused by infection with the filarial parasite *Loa loa*. It is an African disease restricted to the equatorial rain forest regions of Central and West Africa [1], [2], [3], [4]. The limits of its geographical distribution are Benin to the west, Uganda to the east, latitude 10° to the north, and Zambia to the south [5]. The disease is transmitted by *Chrysops* vectors with the major species being *C. silacea* and *C. dimidiata* [6]. The clinical manifestations of loiasis include sub-conjunctival migration of the adult *L.loa* worm, oedema (Calabar swelling) and pruritus [7].

Loiasis has recently emerged as a disease of public health importance, not because of its own clinical manifestations but because of its negative impact on the control of onchocerciasis and lymphatic filariasis in areas of co-endemicity. During the 1990s several patients who harboured a high intensity of *L.loa* infection developed severe adverse neurological reactions after treatment with ivermectin for onchocerciasis in Cameroon [8], [9]. Based on the data for Cameroon, a relationship between the risk of severe adverse reactions and the intensity of *L.loa* infection was established and it was estimated that individuals harboring more than 30000 *L.loa* microfilaria per millilitre of blood (mf/ml) are exposed to a significant risk of serious neurological reactions following ivermectin treatment [8], [9], [10]. The prevalence of high *L.loa* microfilarial loads in endemic communities is directly related to the prevalence of microfilaraemia, and it has been suggested that a microfilarial prevalence of 20% in individuals above the age of 15 years be regarded as the threshold above which there is an unacceptable risk of severe adverse reactions (SAEs) with ivermectin treatment [11].

When the first cases of SAE after ivermectin treatment were reported [9], adequate knowledge was lacking on the geographic distribution of loiasis. Boussinesq and Gardon undertook therefore in 1997 a literature review of available data on the prevalence of *L.loa* microfilaraemia in west and central African regions [12] and identified several zones where loiasis was highly endemic and overlapped with onchocerciasis, e.g. in parts of Cameroon, Gabon and the Democratic Republic of Congo (DRC). However, the available data were limited and there were many areas that were potentially loiasis endemic but for which no local data on *L.loa* infection existed. The published data also had limitations as they were collected over different periods by different researchers using non-standardized diagnostic procedures. Hence there was an urgent need for more detailed, standardized information on the distribution of loiasis in Africa as a basis for operational planning of community directed treatment with ivermectin (CDTi) of onchocerciasis and lymphatic filariasis.

Between 2000 and 2004, environmental risk models were developed and applied for the prediction of loiasis endemicity based on environmental factors (land cover, forest cover and soil type in the initial model [13], and Normalised Difference Vegetation Index or NDVI and elevation in later models [14], [15]) that were favorable for the development of *Chrysops*. These models have helped to clarify the approximate distribution of loiasis endemicity in Africa, but their predictions were not always sufficiently precise [16]. Hence there was a need for local epidemiological surveys in areas that were potentially loiasis endemic and where ivermectin treatment was planned.

The standard parasitological method for the diagnosis of loiasis is the thick blood film. However, this method is not very suitable for large-scale surveys because of its invasiveness and operational constraints. Immunological and molecular methods [17], [18] have been proposed for the diagnosis of loiasis, but have not been sufficiently developed and tested to make them suitable for large-scale epidemiological surveys. There was therefore an urgent need for a non-invasive, simple and rapid method to identify communities in which individuals are at risk of developing SAEs.

A study carried out in Cameroon and Nigeria in 2001, sponsored by the UNICEF/UNDP/World Bank/WHO Special Program for Research and Training in Tropical Diseases (TDR), led to the development of the Rapid Assessment Procedure for Loiasis (RAPLOA) [19], [20]. This method is based on a key clinical manifestation of loiasis, the subcutaneous migration of the adult *L.loa* worm under the conjunctiva of the eye, which is a well-known and highly noticeable experience in loiasis endemic areas. The study demonstrated a close correlation between the prevalence of a history of eye worm and the prevalence *L. loa* microfilaraemia at the community level. Using a threshold of 40%, the prevalence of eye worm history was a good predictor of high-risk communities, i.e. communities where the prevalence of microfilaraemia >20% or where the prevalence of very high intensities of infection (more than 30,000 mf/ml) >2%, with a sensitivity of 100% and specificity ranging from 75 to 90% [20]. The RAPLOA method was subsequently validated successfully in a study in the Republic of Congo and the Democratic Republic of Congo ([21], [22]). The Mectizan Expert Committee and the Technical Consultative Committee of the African Programme for Onchocerciasis Control (APOC) [23] jointly issued in 2004 guidelines for the treatment of onchocerciasis with ivermectin in areas co-endemic for onchocerciasis and loiasis, and recommended that RAPLOA be undertaken to assess the prevalence of *L.loa* before commencing ivermectin distribution in areas suspected, or known, to be endemic for loiasis [24]. APOC subsequently adopted RAPLOA for large-scale loiasis mapping in all potentially endemic areas in APOC countries [25].

This article presents the results of the large-scale implementation of RAPLOA in the 11 APOC countries that were potentially endemic for loiasis (Angola, Cameroon, Central African Republic, Chad, Democratic Republic of Congo, Ethiopia, Equatorial Guinea, Gabon, Republic of Congo, Nigeria and Sudan) and presents a comprehensive map of loiasis as a basis for decision making on ivermectin treatment for the control and elimination of onchocerciasis and lymphatic filariasis in Africa.

## Methods

### Ethics statement

RAPLOA is based on a simple, non-invasive diagnostic method using a short questionnaire, which was developed and validated by the World Health Organization. The RAPLOA survey protocol was reviewed by Technical Consultative Committee of APOC and approved for loiasis mapping in Africa. The surveys in each country were approved by, and undertaken under the authority of, the Ministries of Health of the 11 African countries. Informed consent was obtained from each respondent through a consent procedure as described in the protocol. Each adult above the age of 15 years in a selected household was individually briefed on the objectives of the survey and informed that he/she was free to participate or refuse. Informed consent was orally as many respondents were illiterate. For those who refused to participate, no further questions were asked and no information was recorded. For those who consented, their name, age and years of residence in the community were recorded before proceeding with the RAPLOA interview.

### 1. RAPLOA

The surveys were conducted using the RAPLOA methodology as described in the Guidelines for Rapid Assessment of *L. loa* [26]. This methodology consists of three steps:

- identification of local names for the *L. loa* eye worm using a community-level questionnaire;
- collection of information on the history of eye worm, from adults in the community, using an individual-level questionnaire which has three key questions;
- calculation of the percentage of adults who report a history of eye worm, and, on the basis of this percentage, prediction of the level of *L. loa* endemicity

At the beginning of the RAPLOA survey in each village, the community questionnaire was administered to key informants (village heads, headmasters, schoolteachers, health workers, patent medicine dealers, traditional healers, and women and group leaders) to determine the local names for the eye worm, the population size and the number of households in the community. After administration of the community questionnaire, the geographic coordinates (latitude, longitude, altitude) of the community were collected using a geographical positioning system (GPS) unit in a central point or in front of the house of the village chief.

Households to be included in the survey were then selected randomly. The direction to start was determined by spinning a bottle on the ground and selecting the direction in which the neck of the bottle pointed when it came to a standstill. All adults in the first household, fulfilling the criteria for inclusion - aged 15 years and above, resident in the community for at least 5 years -were interviewed, followed by all adults in the next household, and so on until the required number of 80 individuals per community has been reached. Some villages, notably in Equatorial Guinea, were too small to reach the required sample size and in such villages all adults were interviewed. However, when the total number of adults in the village was less than 20, the village was excluded from the analysis.

The individual questionnaire was designed to elicit responses on experience of eye worm. Three key questions were asked chronologically to collect data on the experience of eye worm. The first question in each interview was “*Have you ever experienced or noticed worms moving along the white of the lower part of your eye ?*”. After recording the response, the interviewer then showed a photograph of the eye worm to each respondent, guided him/her to recognize the worm on the photograph and then asked the second question: “*Have you ever had the condition in this picture?*”. After recording the answer, the interviewer proceeded to ask the third question: “*The last time you had this condition, how many days did the worm last before disappearing?*”.

A respondent was classified as having a history of eye worm when the answers to the first two questions were positive and the duration in the third question was less or equal to 7 days. For each village the percentage of respondents with a history of eye worm was computed to give the prevalence of history of eye worm.

### 2. Sampling

In each country, villages for the survey were selected in areas that were potentially endemic for loiasis. The surveys were conducted in two phases

Phase 1: 2002–2006: During this period, RAPLOA surveys were conducted in areas that were earmarked for ivermectin treatment for onchocerciasis control by APOC and that were located in areas that were potentially endemic for loiasis. Only areas that were meso or hyper endemic for onchocerciasis were targeted.

Phase 2: 2008–2010: with the increasing expansion of NTDs programmes that included the distribution of ivermectin for the elimination of lymphatic filariasis, there was an urgent need by country programmes and partners to have a better knowledge of the distribution of loiasis throughout the African region, including in areas that were not targeted for onchocerciasis control. After it was mandated by its board, the Joint Action Forum, APOC undertook to complete the RAPLOA surveys in the areas outside the onchocerciasis endemic areas not yet covered by RAPLOA surveys.

In every target area, villages were selected with a random spatial sampling procedure to ensure good geographical coverage of the area. The distance between sample villages was around 10 km during phase 1, but when the results of phase 1 showed that the distribution of loiasis was much less localised than initially thought and that there was strong spatial correlation in eye worm prevalence over distances up to 100–200 km, the distance between sample villages was gradually increased to about 25 km during the last round of surveys of phase 2. Villages were selected using the Healthmapper software and data base (http://www.who.int/health_mapping/tools/healthmapper) or a 1∶200,000 scale local paper map of the area.

### 3. Data processing

Data entry was mostly performed by the survey teams at country level using Microsoft Excel@ but sometimes at APOC headquarters using SPSS data entry builder@. Only aggregate village level data were entered: total population, number interviewed, number and percentage with eye worm history and location information, i.e. GPS readings of latitude and longitude, name of village, names of all administrative levels. When RAPLOA results were received at APOC headquarters, systematic data checking was undertaken including the validation of geographical coordinates (latitude, longitude) of all surveyed villages using geographical information system software (Atlas*GIS^@^, ArcGIS@). Where available, the geographic coordinates of survey villages were compared to the coordinates found in the *GADM database* of the Global Administrative Areas (http://www.gadm.org). All RAPLOA data were then integrated into a master database in Microsoft Access@ at APOC headquarters.

### 4. Spatial analysis

The survey data were first analyzed using SPSS version 15 (www.spss.com) to generate summary tables and bar charts on the survey activities by country and year. The geographical information system software ArcGIS version 10 (ESRI Inc., Redlands, USA) was used for spatial analysis of the RAPLOA data. The prevalence of history of eye worm for each village was submitted to a logit transformation. The transformed prevalence data were then analyzed through a geostatistical method called kriging using the Geostatistical Analyst Extension of ArcGIS v10. The kriging analysis involved variography to determine the spatial correlation pattern in the survey data and a process of weighted spatial smoothing to predict the distribution of the logit prevalence throughout the surveyed area. Kriging gives a predicted prevalence at any location, but with poor precision at large distances from the sampled locations. We therefore defined the “surveyed area” pragmatically as the area where the local prediction standard error was smaller than, or equal to, the average standard error obtained in the cross validation analysis of the difference between predicted and observed logit prevalences for the surveyed villages. This definition ensured that the “surveyed area” covers all surveyed villages but does not extend beyond a distance of 40 to 100 km from the nearest surveyed village. For each location in the surveyed area, the predicted probability that the true prevalence exceeds 40% was estimated by calculating Z = {(logit(0.4)−M)/S, where M is the local predicted logit prevalence and S the prediction standard error, and using the normal distribution to determine the corresponding probability. The predicted logit prevalences were back transformed to the original scale to produce prevalence and probability contour maps for the surveyed area.

The contour map was also used to estimate the proportion of each country surface that was mapped by RAPLOA, and to divide the mapped surface into 4 loiasis endemicity classes with prevalence of eye worm 0–4%; 5–19%; 20–39% and > = 40%. The rural population in each class was tentatively estimated as the total rural population for the country multiplied by the proportion of country surface falling in that class, assuming a uniform distribution of the rural population in the country. These estimates will be refined when a detailed population density map for the rural population of Africa becomes available. The boundaries and the surface (in square kilometers) of the 11 African countries were obtained from the GADM database of Global Administrative Areas (http://www.gadm.org). The total rural population for each of the 11 countries was extracted from the database of the United Nations department of Economic and Social Affairs (http://esa.un.org/unpd/wpp2008/tab-sorting_population.htm).

## Results

RAPLOA surveys were undertaken in a total of 4798 villages in the 11 APOC countries that were known or suspected to be endemic for loiasis (see table 1). In 10 countries, the RAPLOA surveys confirmed the presence of loiasis and in each of these countries there were high risk villages where 69% to 100% of those interviewed reported a history of eye worm. In Equatorial Guinea and Gabon, eye worm was reported from all surveyed villages. Only in Ethiopia did none of the respondents report a history of eye worm.

**Table 1:** Number of villages surveyed and number of people interviewed in 11 APOC countries.

Country	No. villages surveyed	No. of people interviewed	Mean no. interviewed per village	No. interviewed who had history of eye worm	Percentage with eye worm history per village		
					Minimum	Median	Maximum
ANGOLA	222	18,589	83.7	2,822	0.0	5.6	98.8
CAMEROON	812	66,996	82.5	28,622	0.0	0.0	98.8
CAR	173	13,874	80.2	6,310	0.0	47.5	95.6
Chad	111	8,876	80.0	913	0.0	0.0	87.5
CONGO	195	14,666	75.2	6,647	0.0	50.0	100.0
DRC	2,516	199,766	79.4	42,710	0.0	13.8	86.3
EQUATORIAL GUINEA	84	4,907	58.4	3,208	11.8	70.9	100.0
ETHIOPIA	28	2,240	80.0	0	0.0	0.0	0.0
Gabon	65	5,015	77.1	3,263	23.6	66.3	95.0
NIGERIA	381	30,106	79.0	5,708	0.0	18.8	69.5
SUDAN	211	16,540	78.4	3,138	0.0	10.0	93.0
Total	4,798	381,575	79.6	103,341	0.0	20.8	100.0

**Figure 1:**
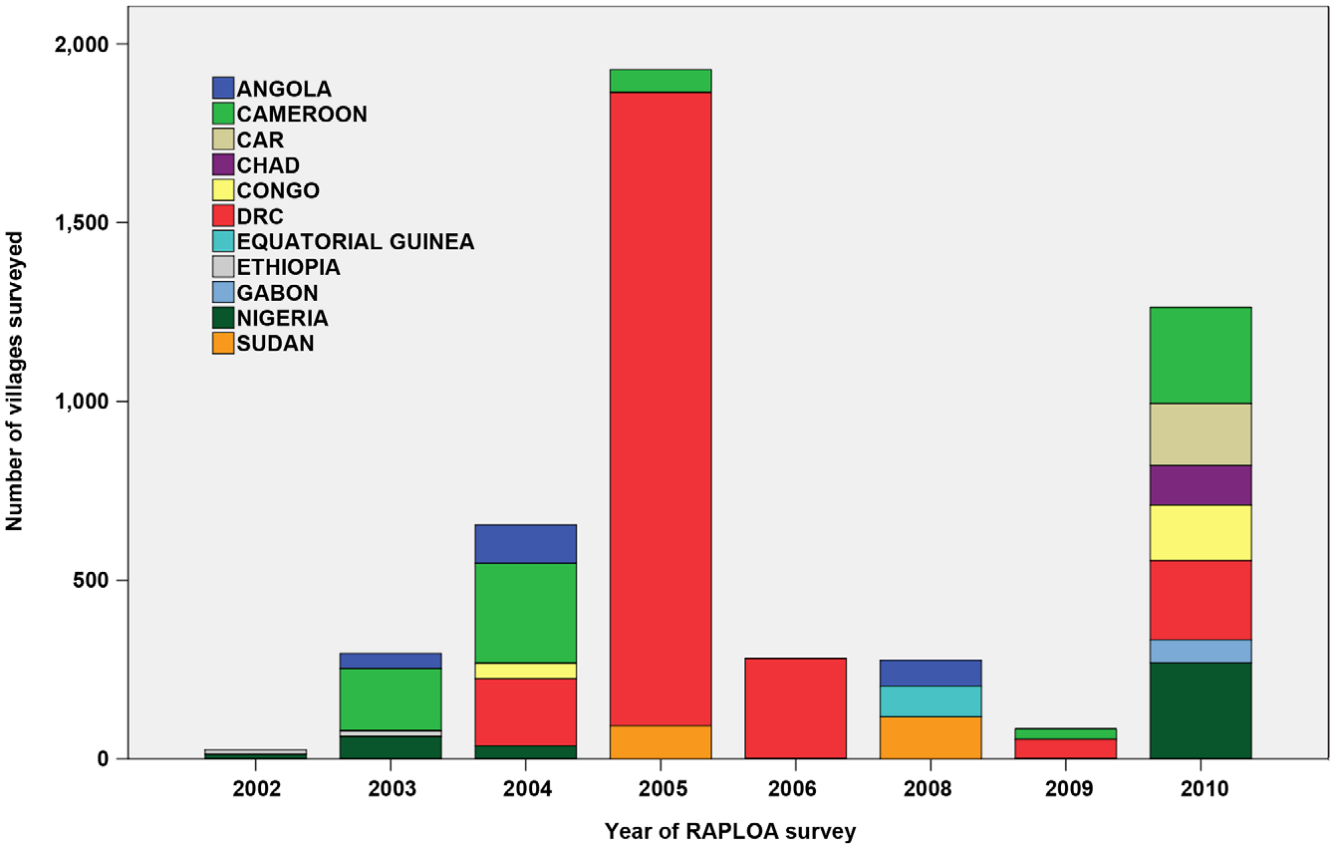
Number of villages surveyed for RAPLOA by country and year.

The surveys were done in two major phases between 2002 and 2010 (figure 1). The first phase from 2002 to 2006 was triggered by the occurrence of SAEs after ivermectin treatment in Cameroon and DRC, and the urgent need of CDTi projects in these two countries to understand the local endemicity of loiasis and the corresponding risk of SAEs. The need for such information was especially great in DRC where a large number of CDTi projects were to be launched around that time. A major survey effort was therefore undertaken in 2005 during which as many as 1,771 RAPLOA surveys were done in DRC alone. The second major survey effort was in 2010 after APOC undertook to complete the RAPLOA mapping in Africa, including in areas not targeted for onchocerciasis control but that were of importance for lymphatic filariasis elimination with ivermectin treatment. This second effort filled several remaining gaps in the survey coverage of the total area in Africa where loiasis is potentially endemic.

**Figure 2:**
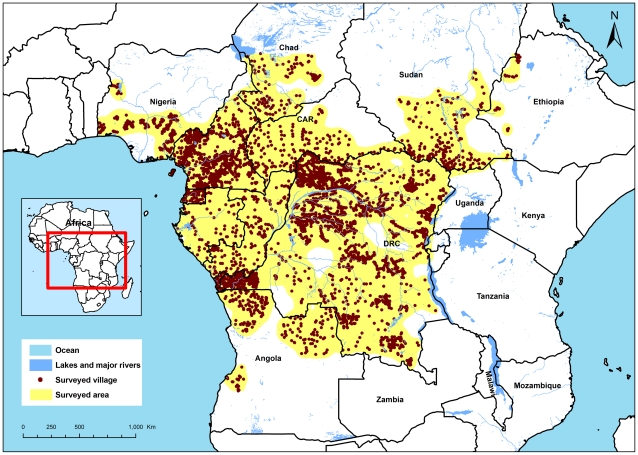
Geographical distribution of RAPLOA surveyed villages in 11 APOC countries.

The locations of the survey villages and the boundaries of the “surveyed area” are shown in figure 2. The geographic distribution of survey villages is not uniform and in Cameroon and DRC there are some areas with a heavy concentration of surveyed villages. This reflects the intensified efforts of 2003 to 2005 in response to urgent survey needs of specific CDTi projects in those areas. In subsequent years, and particularly in 2010, a grid-based sampling method was introduced to select RAPLOA survey villages at more regular distances to ensure better spacing of the sample. Altogether, the RAPLOA survey villages cover a vast area of some 2500 km×3000 km centred on the heartland of loiasis in central equatorial Africa.

**Figure 3:**
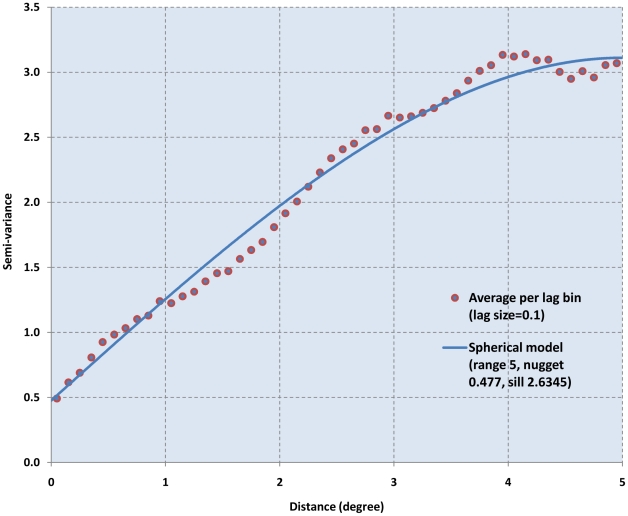
Semi-variance of the prevalence of history of eye worm in relation to distance between survey villages.

The spatial analysis of the RAPLOA data showed a strong spatial correlation pattern. This is illustrated in the variogram in figure 3 which shows the semi-variance, a measure of the variation in prevalence data in relation to the distance between survey villages. At short distances, the semi-variance is small, indicating that villages that are located closely together tend to have similar prevalences of history of eye worm. With increasing distance, the semi-variance increases and consequently the spatial correlation declines. This spatial correlation pattern has been modeled as shown by the solid line in figure 3 (spherical model with range 5, nugget 0.477 and sill 2.6345). This model was subsequently used in a kriging analysis of the RAPLOA data to produce, through a process of spatial smoothing, a map of the prevalence of eye worm history throughout the surveyed area.

**Figure 4:**
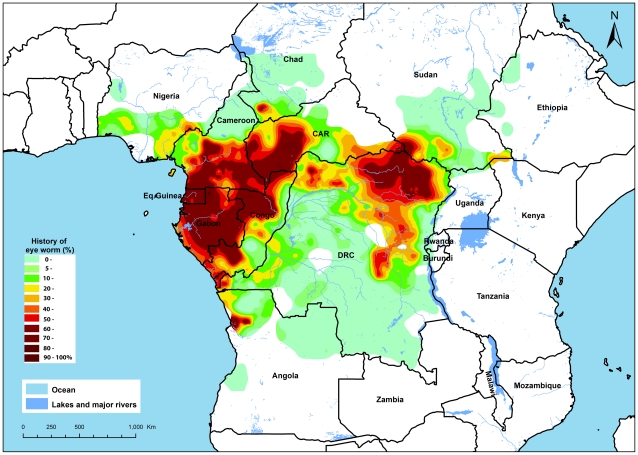
Map of the estimated prevalence of eye worm history in Africa.

Figure 4 shows the results of the kriging analysis. This map provides the best estimate of the geographic distribution of loiasis based on the RAPLOA data. The main geographic pattern is clear. There are two zones of highly endemic loiasis: a western zone that comprises the totality of the continental part of the Equatorial Guinea and Gabon, Cameroon south of 6°N, and parts of the Republic of Congo, the Central African Republic and Chad. This western zone also comprises the Mayombe forest in the west flank of the Bas-Congo province in the DRC and the Cabinda and west of Bengo provinces in Angola. The second hyper-endemic zone is mainly made up of the North-Eastern part of the Democratic Republic of Congo. It has its epicenter in the Province Orientale with extensions towards the Equateur province in the west, Maniema province in the south and Sudan in the north-east. There are also vast areas where there is no loiasis or where its endemicity is very low, e.g. in most of DRC, north Cameroon and large sections of Angola, Nigeria, Chad and Sudan. In between there are some intermediate zones where the estimated prevalence of eye worm history ranges between 20 and 40%.

**Figure 5:**
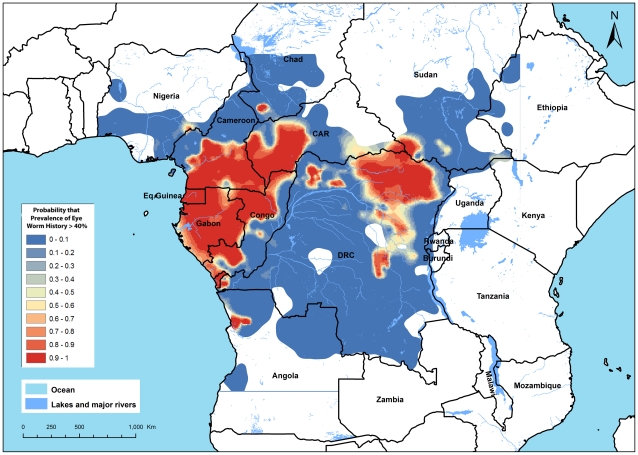
Map of the predictive probability that the local prevalence of eye worm history exceeds 40%.

The estimates given in figure 4 involve statistical uncertainty which is important to take into account, especially around the policy threshold value of a prevalence of 40% eye worm history. Figure 5 therefore provides a map of the predicted probability that the local prevalence of eye worm history exceeds 40%. In most of the surveyed area, there appears to be little uncertainty and the probability that the prevalence exceeds the threshold is whether very high (>0.9) or very low (<0.1). Hence, these results strengthen the above conclusion with respect to areas with very high and very low endemicity. However, in some intermediate areas, the results are less clear-cut. In such areas it will be important to inspect the available data in greater detail to assess the operational implications of the RAPLOA findings for local ivermectin treatment programs.

**Figure 6:**
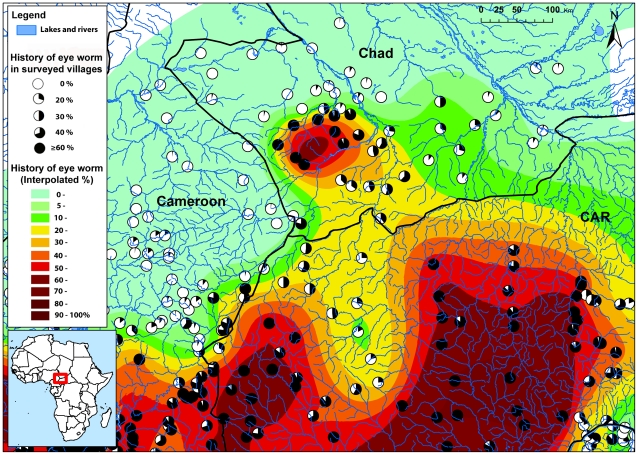
map of the estimated prevalence of eye worm history in the border area between Chad, Central African Republic and Cameroon.

As an example of this process, figure 6 provides a detailed map of the border area of Chad, Cameroon and the Central African Republic (CAR). In south Chad the RAPLOA data revealed the existence of a previously unknown focus of hyperendemic loiasis. Across the border in CAR the spatial analysis showed a vast area of hyperendemic loiasis where the prevalence of eye worm was very high for all surveyed villages. In the centre of the map, between these two hyperendemic areas, there is a zone for which the RAPLOA prevalence data are between 20% and 40% and which the kriging analysis has classified as intermediate and below the risk threshold of 40%. Nevertheless, being so close to two highly endemic zones, it might be prudent in such a borderline area to take the same precautionary measures as in the surrounding highly endemic areas when implementing ivermectin treatment. Such a strategy might also be operationally more convenient if the intermediate and high endemicity groups of villages fall under the same implementation unit of the health system.

Table 2 shows the result of an attempt to estimate the population at risk in the different countries using the RAPLOA map. Five countries, Cameroon, Congo, DRC, Equatorial Guinea and Gabon, have been nearly completely mapped for loiasis. The other six countries were only partly covered by RAPLOA surveys. Some regions were purposely excluded because they were known to be loiasis free, e.g. the desert regions of Chad and Sudan. The North East of CAR and the bordering area in Sudan could not be surveyed because of security reasons while the mapping of Angola is not yet complete. However, the vast majority of potentially loiasis endemic areas in Africa have been mapped.

**Table 2:** Estimated areas and population at risk for loiasis in 11 APOC countries.

						Percentage of mapped area by Prevalence of eye worm				Rural population (×1000) by Prevalence of eye worm			
Country	Rural population (×1000)	Area country (km2)	Area mapped (km2)	% mapped	Rural population in mapped area (×1000)	0%–4.9%	5%–19.9%	20%–39.9%	40%– 100%	0%–4.9%	5%–19.9%	20%–39.9%	40%–100%
Angola	7 881	1 252 421	427 714	34%	2 691	66,6	20,9	6,8	5,7	1 792	563	183	153
Cameroon	8 303	466 307	451 857	97%	8 046	27,8	11,2	10,9	50,2	2 234	900	873	4 038
CAR	2 751	621 499	445 381	72%	1 971	4,8	20,8	29,1	45,3	95	410	573	893
Chad	8 328	1 168 002	326 493	28%	2 328	80,0	11,9	6,0	2,1	1 862	276	141	49
Congo	1 424	345 430	344 685	100%	1 421	3,5	21,2	22,8	52,5	497	301	324	746
DRC	43 940	2 337 027	2 215 074	95%	41 647	43,3	22,8	16,2	17,7	18 017	9 510	6 743	7 377
Eq. Guinea	418	27 085	26 950	99%	416	0,0	0,0	7,6	92,4	0	0	31	384
Ethiopia	70 818	1 132 328	82 460	7%	5 157	100,0	0,0	0,0	0,0	5 157	0	0	0
Gabon	210	261 689	260 764	100%	209	0,0	0,1	2,6	97,3	0	0	6	204
Nigeria	79 441	912 039	278 233	31%	24 235	20,6	54,8	23,5	1,0	4 997	13 281	5 703	254
Sudan	25 871	2 490 410	511 017	21%	5 309	64,3	18,4	12,4	4,8	3 416	977	658	257
**Grand Total**	**249 385**	**11 014 237**	**5 370 628**		**93 430**	**40%**	**20%**	**15%**	**25%**	**37 621**	**26 218**	**15 235**	**14 357**

Table 2 shows a breakdown of the mapped area by loiasis endemicity level. Equatorial Guinea and Gabon are the most endemic countries where nearly the whole area falls into the high risk category with more than 40% RAPLOA prevalence. In DRC, only 18% of the area falls into this category but because of its much larger population, this translates into an estimated population of 7.4 million people living in high risk areas. In terms of population at high risk, Cameroon comes second with 4 million people. DRC and Cameroon together account for 80% of the estimated 14.4 million people living in high risk areas.

## Discussion

The RAPLOA surveys represent a major effort of the African Programme for Onchocerciasis Control in response to a serious operational challenge for onchocerciasis control and lymphatic filariasis elimination. Within two periods of a few years, thousands of rapid assessment surveys were done in order rapidly to generate the local data on loiasis endemicity levels that were needed for planning of ivermectin treatment in potential loiasis areas. The surveys were undertaken by the Ministries of Health in the affected countries, with technical and financial support from APOC. The technical support, provided by a group of African experts, has also contributed to strengthening national capacity for epidemiological evaluation and surveillance.

Initially, the RAPLOA surveys targeted areas where CDTi projects were planned and where information on loiasis endemicity was urgently needed. These CDTi projects needed to understand the local risk of adverse reactions to guide decision making on appropriate measures for monitoring and management of possible SAEs in accordance with the requirements of the Mectizan Donation Program. As the RAPLOA survey data accumulated, the beginning of a loiasis map began to emerge. The final round of surveys in 2010 filled most of the remaining gaps in survey coverage and a comprehensive evidence-based map of loiasis is now available that covers most of the potentially loiasis endemic area in the world. The only large areas that remain to be mapped are a border area between CAR and Sudan, which has a low population density but is likely to be highly endemic for loiasis, and much of Angola, where loiasis may not be widespread. Beyond the APOC countries, loiasis is rare; one focus of low endemicity is known in south Benin, and a few sporadic cases reported from Zambia [12].

The resulting map of the prevalence of eye worm history is unique and provides the first global map of loiasis based on actual survey data. The map shows a clear geographic distribution of loiasis with two zones of hyper-endemicity, large areas that are free of loiasis or of low endemicity, and several borderline or intermediate zones including one zone in north-west DRC that bridges the two hyper-endemic zones. The implications for ivermectin treatment are evident: in the hyper-endemic zones there is a high risk of SAEs and special precautionary measures are required in accordance with the MDP guidelines [16], [24]. For the loiasis-free and low endemic areas no special measures are required and ivermectin treatment can be implemented without risk. The intermediate zones will generally require more detailed assessment of the available data, as demonstrated above by the example for South Chad, in order to support local decision-making on ivermectin treatment. APOC will therefore make the necessary detailed maps available to endemic countries and their partners in onchocerciasis control and lymphatic filariasis elimination, and publish these maps on its website (www.who.int/apoc). The data for Chad also provide a good example of important new information that has become available through the RAPLOA surveys. It is always been assumed on the basis of few data that loiasis was rare and of very low endemicity in Chad [12], [27], [28], [29] and the discovery of a hyperendemic focus in the southern part of this country was a surprise. Similarly, RAPLOA has clarified the distribution of loiasis in the Central African Republic and large parts of Congo and DRC for which hardly any information was available previously.

In addition to providing important new information on the distribution of loiasis, the RAPLOA surveys have confirmed the continued existence of known loiasis foci in several countries ([12]. In Cameroon the previously documented *L. loa* foci in the south region [30], the centre region [31], Adamaoua region [32], Littoral region [30], [33], and the south-west region [1] have all been confirmed. New foci have been revealed in the North West and Adamaoua regions situated in savannah areas that were not known to be endemic for loiasis. In Nigeria, the RAPLOA surveys indicate that the level of endemicity of loiasis is relatively low. The most affected areas are south of latitude 6°N, between the Niger delta and the border with Cameroon, which is in conformity with previous knowledge [34], [35], [36]. For the Central African Republic, hardly any data were available [37] but the RAPLOA surveys have shown that loiasis is highly endemic in the south west and south east of the country. In the Democratic Republic of Congo, the well known highly endemic focus of Mayombe in the most western part of Bas-Congo province and of Ueles in the North-eastern part of province Orientale ([38], [39] have also been confirmed.

The main endemicity pattern as shown on the RAPLOA map is broadly similar to the pattern on the map produced by Thomson et al [13] using an environmental risk model which also shows high endemicity in the West and the East, and a zone of lower endemicity in between. However, there are also major discrepancies between the two maps. The environmental risk map predicts the highest endemicity in Congo and south-west DRC, but the RAPLOA survey showed that endemicity levels in both areas were very low. Conversely, the map of Thompson et al suggests that the Central African Republic is largely loiasis free while the RAPLOA surveys showed a very high endemicity level throughout nearly half the country. Hence the environmental risk models, though useful for showing general trends, are not reliable enough for use in operational decision making for ivermectin treatment. Diggle et al [15] subsequently developed a spatial statistical model that incorporated the environmental risk variables NDVI and elevation, for the analysis of epidemiological survey data on the prevalence of *L.loa* microfilaraemia. The application of this model to prevalence data for Cameroon showed a significant improvement over the Thompson model. However, a comparison with the RAPLOA map showed that model predictions at more than 100 kilometres from the nearest survey village were sometimes also very inaccurate. One possible explanation is that NDVI and elevation have only limited predictive value on their own, as suggested by the low correlation between these environmental variables and the prevalence of MF [14], [15]. Using the results of a calibration analysis of the relationship between the prevalence of RAPLOA and the prevalence of MF, the RAPLOA data are now being incorporated into the spatial statistical model in order to enhance its predictive value.

On the basis of the RAPLOA results, it is tentatively estimated that some 14.4 million people live in high risk areas where the estimated prevalence of eye worm history is greater than 40%, and 15.2 million in intermediate areas with estimated eye worm prevalences between 20 and 40%. The number of people at high risk varies considerably between countries. Nearly the whole country of Gabon is classified as high risk, and represents a large proportion of the total high risk area in Africa, but because of the low population density in Gabon it represents less than 2% of the total high risk population. DRC with 7.4 million and Cameroon with 4 million represent together 80% of the estimated total population at high risk.

Not all highly endemic loiasis areas overlap with the geographic distribution of onchocerciasis or lymphatic filariasis but in most of the surveyed countries there is considerable overlap and thus a significant risk of SAEs with ivermectin treatment. The map of the prevalence of eye worm therefore provides critical information for ivermectin treatment programs among millions of people in Africa. This information comes particularly timely for lymphatic filariasis elimination for which loiasis has been a major barrier in Central Africa [40] but which can now go ahead in the many areas where loiasis endemicity is low or nil.
